# Three-Dimensional Changes in Natural Head Position After Bimaxillary Orthognathic Surgery: A Laser-Based Facial Scan Superimposition Study in Skeletal Class II and Class III Patients

**DOI:** 10.3390/jcm15166434

**Published:** 2026-08-20

**Authors:** Vincenzo Abbate, Gianluca Renato De Fazio, Francesco Maffia, Eutilia Manzo, Serena Trotta, Marco Friscia, Maria Esposito, Stefania Troise, Giovanni Salzano, Paola Bonavolontà, Fabio Maglitto, Giovanni Dell’Aversana Orabona

**Affiliations:** 1 Maxillofacial Surgery Unit, Department of Neurosciences, Reproductive and Odontostomatological Sciences, University of Naples “Federico II”, Via Sergio Pansini 5, 80131 Naples, Italy; vincenzo.abbate@unina.it (V.A.); eutilia.manzo@libero.it (E.M.); serenatrotta99@icloud.com (S.T.); friscia.marco5@unina.it (M.F.); stefania.troise@unina.it (S.T.); giovannisalzanomd@gmail.com (G.S.); paola.bonavolonta@unina.it (P.B.); fabio.maglitto@aoufedericoii.unina.it (F.M.); giovanni.dellaversanaorabona@unina.it (G.D.O.); 2 Instituto Português da Face, 1500-315 Lisbon, Portugal; 3 PhD Program, Faculty of Medicine, University of Lisbon, 1649-028 Lisbon, Portugal

**Keywords:** natural head position, orthognathic surgery, 3D facial scanning, cranio-cervical posture, surface superimposition, skeletal malocclusion, virtual surgical planning

## Abstract

**Background/Objectives:** Natural head position (NHP) is an important reference for orthodontic and orthognathic assessment. Although postoperative changes in head posture have been reported previously, simultaneous three-dimensional changes across yaw, pitch, and roll in different skeletal classes remain incompletely characterized. This exploratory study quantified six-month changes in three-dimensional head orientation and compared their direction and magnitude between skeletal Class II and Class III patients. **Methods:** Fifty-four adults (17 Class II, 37 Class III) undergoing Le Fort I osteotomy and bilateral sagittal split osteotomy were evaluated preoperatively (T0) and 6 months postoperatively (T1). Standardized 3D facial surface acquisitions were obtained using a calibrated structured-light scanner and a fixed external Cartesian laser reference. T0 and T1 datasets were registered using stable upper-facial landmarks (nasion and bilateral exocanthia), and changes in yaw, pitch, and roll were calculated within the common external reference system. **Results:** Statistically significant T0–T1 changes were observed across all three axes in both skeletal groups (*p* < 0.01). Class II patients showed mean changes of +2.28° in yaw, −1.30° in pitch, and +2.89° in roll, whereas Class III patients showed +4.61°, +1.96°, and +4.04°, respectively. Between-group differences were significant for yaw, pitch, and roll, with an opposite mean pitch direction and larger mean yaw and roll changes in Class III. **Conclusions:** At six months, this retrospective cohort demonstrated direction-specific changes in three-dimensional head orientation. Because repeated acquisitions, study-specific observer-reliability and measurement-error analyses, an untreated comparator, an independent validation cohort, and functional or patient-reported outcomes were not available, the findings should be interpreted as exploratory measurements rather than evidence of physiological normalization, neuromuscular adaptation, or clinical benefit. Formal prospective validation is required before the workflow can support predictive or patient management recommendations.

## 1. Introduction

The natural head position (NHP) has long been regarded as a cornerstone of orthodontic and orthognathic assessment, providing a physiologically meaningful and clinically reproducible reference for diagnosis and treatment planning. Defined as the neutral orientation of the head when an individual stands upright and directs the gaze toward a distant point on the horizontal plane, the NHP reflects the habitual balance of craniofacial posture in everyday life [1,2]. This property makes it particularly valuable for cephalometric analysis and three-dimensional (3D) surgical planning, where accuracy and consistency are crucial [3,4]. Unlike intracranial reference systems, which may suffer from variability and limited clinical relevance, the NHP has demonstrated superior stability and reproducibility over time [3,5,6]. Longitudinal investigations have consistently shown minimal method errors, often within 2–3°, even across follow-up intervals extending to 5 and 15 years [2,3]. Such evidence underscores the reliability of the NHP as a stable external reference, reinforcing its central role in both clinical practice and research [5,6].

Cranio-cervical posture is associated with underlying craniofacial morphology. An obtuse cranio-cervical angle has been correlated with increased lower anterior facial height and a steep mandibular plane, whereas an acute angle is more commonly reported in patients with reduced vertical facial dimensions and a flatter mandibular plane [7]. These associations have led to the hypothesis that skeletal disharmony may be accompanied by compensatory changes in head posture. Postoperative changes in NHP have been described using lateral cephalograms, cone-beam computed tomography (CBCT), stereophotogrammetry, and other three-dimensional approaches [8,9,10]. Such observations indicate that head orientation may change after orthognathic treatment, but the biological mechanisms underlying these changes, including possible neuromuscular, cervical, or airway-related pathways, cannot be inferred from head-orientation measurements alone.

Previous studies have demonstrated postoperative changes in head posture using cephalometric, CBCT-based, stereophotogrammetric, and other three-dimensional approaches. The present study therefore does not claim that postoperative modification of NHP is itself a novel observation. Its incremental contribution is the simultaneous exploratory quantification of yaw, pitch, and roll in skeletal Class II and Class III patients within a single standardized acquisition and analytical framework. Accordingly, this exploratory study aimed to quantify six-month changes in yaw, pitch, and roll of NHP and to compare their direction and magnitude between skeletal Class II and Class III patients. The findings are intended to inform the interpretation of head orientation during virtual surgical planning and postoperative assessment, rather than to prescribe a specific planning protocol, define recommended aesthetic outcomes, or establish a biological mechanism.

## 2. Materials and Methods

### 2.1. Study Design and Setting

This single-centre retrospective observational pre-post cohort study was conducted at the Maxillofacial Surgery Unit, University of Naples “Federico II”. Consecutive institutional records were screened for patients who underwent bimaxillary orthognathic surgery between January 2020 and December 2023. Throughout the analysed cohort, facial-scan acquisition was performed using the same scanner, external laser-reference system, acquisition environment, patient-positioning procedure, and trained operator, thereby maintaining methodological homogeneity across the study period. No untreated comparator or independent validation cohort was available. The study was designed, conducted, and reported in accordance with the STROBE guidelines for observational studies and adhered to the principles of the Declaration of Helsinki.

### 2.2. Participants

Patients were eligible for inclusion if they met all of the following criteria: age ≥ 18 years at the time of surgery; availability of paired preoperative and postoperative NHP records obtained via laser-referenced 3D facial scanning; and treatment by bimaxillary osteotomy (Le Fort I and bilateral sagittal split osteotomy) with or without genioplasty. Exclusion criteria were syndromic craniofacial anomalies, history of facial trauma, previous facial aesthetic surgery or procedures, use of neuromuscular blocking agents within six months prior to imaging, and cervical-spine skeletal abnormalities evident on radiographic examination. Craniofacial asymmetries associated with congenital or syndromic malformations were excluded. Mild asymmetries commonly associated with skeletal malocclusion were retained because they represent typical orthognathic presentations; stable upper-facial landmarks were selected to reduce the influence of surgically altered regions during registration. Malocclusion was classified as skeletal Class II or Class III on the basis of cephalometric ANB angle and clinical assessment. No a priori sample-size calculation was used to determine recruitment because this retrospective study included all consecutive eligible patients with complete paired records during the predefined study period. To characterize the precision and magnitude of the observed effects, results are reported with 95% confidence intervals and standardized effect sizes in addition to *p*-values. The smaller Class II subgroup reduces estimate precision, and the subgroup findings should therefore be interpreted as exploratory rather than predictive.

### 2.3. Surgical Procedures

All operations were performed under general endotracheal anaesthesia following standardized orthognathic protocols. Each patient underwent a Le Fort I maxillary osteotomy combined with a bilateral sagittal split osteotomy (BSSO) of the mandible; genioplasty was added when indicated for chin harmonization. Osteotomies were performed using either piezosurgical or rotary instruments, and rigid internal fixation was achieved with titanium miniplates and screws. Intraoperative occlusal positioning was guided by intermediate and final splints fabricated from preoperative virtual surgical planning [11,12]. Transverse maxillary width, when addressed, was managed according to the unit’s standardized protocol [13].

### 2.4. Three-Dimensional Data Acquisition

**Figure 1:**
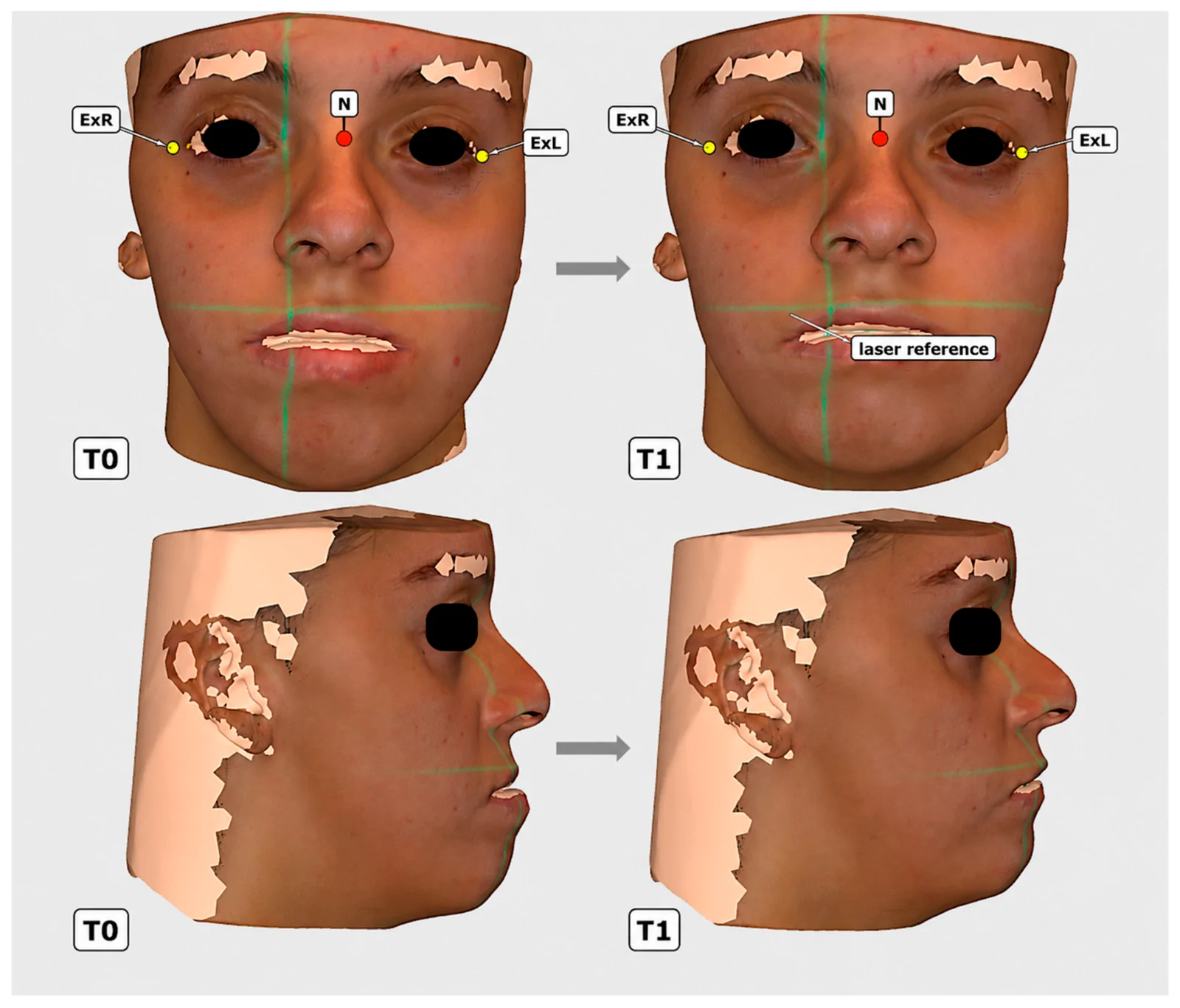
Standardized preoperative (T0) and 6-month postoperative (T1) 3D facial acquisitions obtained with the EinScan Pro HD structured-light system and the Kiprim LV1D cross-line laser reference. Frontal and lateral views are shown. The stable upper-facial landmarks used in the analytical workflow are indicated as nasion (N), right exocanthion (ExR), and left exocanthion (ExL). The projected laser reference provides the fixed environmental reference used during serial acquisition.

Three-dimensional facial surface data were obtained preoperatively (T0) and 6 months postoperatively (T1). Facial surfaces were acquired with an EinScan Pro HD structured-light scanner (Shining 3D, Hangzhou, China). Before data acquisition, the system was calibrated according to the institutional acquisition protocol, which specified a minimum calibration accuracy of 1 μm. The system was selected because its acquisition workflow was compatible with the external reference-based protocol used in this study; the study was not designed as a comparative evaluation of alternative scanners or software. A Kiprim LV1D cross-line laser system (Kiprim, Shenzhen, China) projected a fixed environmental Cartesian reference frame orthogonal to the floor. The projected cross-line laser references were recorded directly within each 3D dataset, thereby preserving the same external Cartesian reference at T0 and T1 and enabling objective comparison of head orientation relative to a common environmental frame (Figure 1).

All T0 and T1 acquisitions were performed during the morning as part of the standardized acquisition protocol in order to maintain a consistent time-of-day window and minimize potential short-term postural variability, including possible fatigue-related changes over the course of the day. All acquisitions were performed in the same room, with the same scanner, laser-reference system, lighting conditions, patient-positioning procedure, and trained operator. Patients stood upright and were instructed to adopt a self-balanced natural head position while gazing at a distant point at eye level, without external support or physical manipulation. Before each acquisition, the operator visually confirmed a stable self-balanced NHP and correct alignment of the projected Cartesian laser reference. Repeated complete facial scans were not routinely obtained; only the accepted acquisition was retained for analysis. Consequently, intra-subject acquisition repeatability could not be quantified directly.

Fixed orthodontic appliances were present in all patients at T0. At T1, brackets remained present only in patients who had not yet completed postoperative orthodontic treatment, whereas they had been removed in patients who had completed the orthodontic phase. This difference in appliance status was not expected to materially affect the rotational analysis because neither the dentoalveolar region nor the orthodontic appliances were used for registration or angle calculation. After confirmation of NHP, the operator performed a circumferential facial scan, capturing frontal, lateral, and oblique views in a continuous sequence while moving around the patient at a constant distance according to the manufacturer’s protocol. Completed scans were visually inspected for artefacts, image completeness, and overall acquisition quality before being accepted for processing.

### 2.5. Data Processing and Superimposition

**Figure 2:**
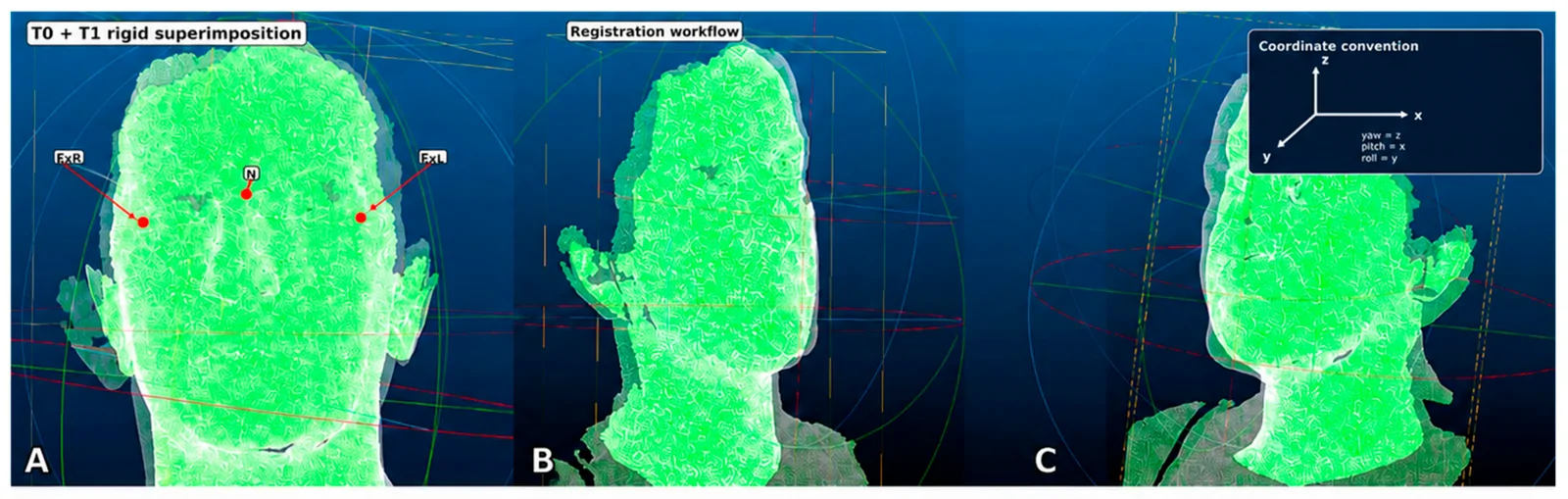
Rigid registration workflow for the preoperative (T0) and postoperative (T1) facial scans. The darker surface represents T0 and the lighter surface represents T1. (**A**) Frontal view of the rigidly superimposed T0 and T1 facial surfaces, showing the predefined stable upper-facial landmarks: nasion (N), right exocanthion (ExR), and left exocanthion (ExL). (**B**) Lateral view of the rigid registration workflow and spatial relationship between the T0 and T1 facial surfaces. (**C**) Lateral view of the registered datasets showing the Cartesian coordinate convention: x, medio-lateral; y, antero-posterior; z, vertical; yaw, rotation about z; pitch, rotation about x; and roll, rotation about y. The coloured lines visible in the panels are software-generated reference and registration guides used for spatial orientation and do not represent quantitative results. The figure illustrates the registration workflow and spatial relationship between the serial datasets and is not intended as a quantitative assessment of registration accuracy.

**Figure 3:**
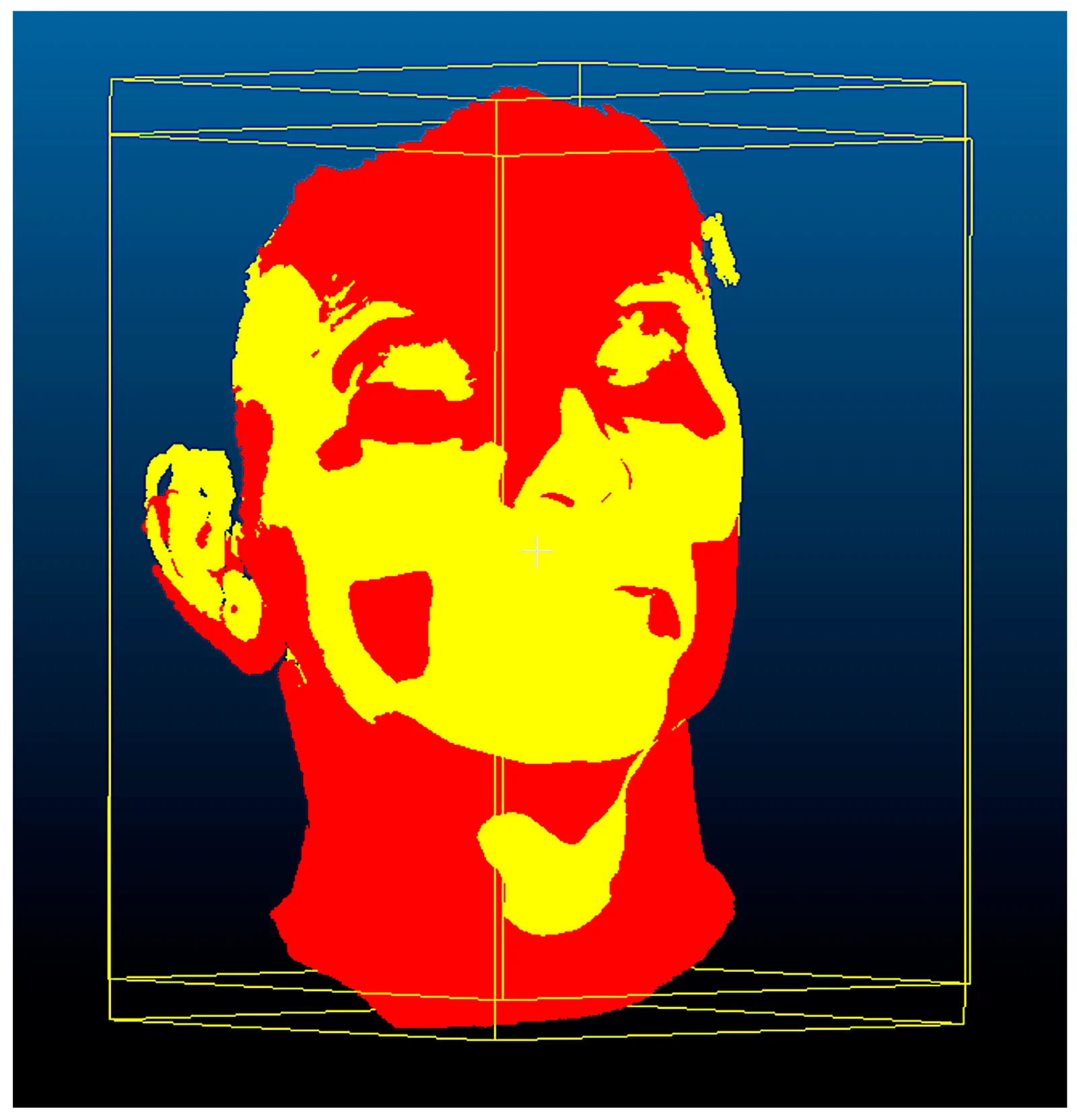
Qualitative colour-coded visualization of craniofacial surface displacement following rigid registration of the T0 and T1 scans. The map is provided solely to illustrate the spatial pattern of surface differences after registration; it should not be interpreted as a measurement-error analysis, a quantitative assessment of registration accuracy, or validation of the registration method.

The 3D mesh files were processed in EXScan Pro (v4.0; SHINING 3D, Hangzhou, China) for mesh quality control, surface cleaning, and OBJ export. The manufacturer’s software was not used to calculate head rotations. For each scan, the nasion and bilateral exocanthia were manually identified as predefined stable upper-facial landmarks. Preoperative (T0) and postoperative (T1) meshes were then aligned in CloudCompare (v2.6) using rigid surface-based registration (Figure 2). A standard iterative-closest-point (ICP) algorithm was applied following initial landmark-based alignment. Each registration was visually inspected to exclude obvious processing errors before rotational measurements were extracted. A qualitative colour-coded surface displacement map following rigid registration is shown in Figure 3. The use of upper-facial reference landmarks was intended to reduce the influence of the surgically altered middle and lower facial regions; however, this procedural standardization should not be interpreted as formal validation of registration accuracy or observer reliability.

To quantify head reorientation, a head-fixed Cartesian coordinate system was constructed for each acquisition from the three upper-facial landmarks. The origin was placed at the nasion; the medio-lateral (x) axis was defined by the line joining the bilateral exocanthia; the vertical (z) axis was oriented orthogonal to the floor in accordance with the projected laser reference; and the antero-posterior (y) axis was obtained as the cross-product of the other two, yielding an orthonormal basis. Because the external cross-line laser reference was retained within every scan, NHP at each time point could be expressed as the orientation of the head-fixed frame relative to the same environmental frame. The postural change was computed as the relative rotation matrix R = R(T1)·R(T0)^−1^ and decomposed into sequential Euler rotations, yielding yaw (rotation about z), pitch (rotation about x), and roll (rotation about y), as illustrated in Figure 2. Neither the dentoalveolar region nor orthodontic appliances contributed to the definition of the reference landmarks or rotational axes.

### 2.6. Outcome Measures

The primary outcomes were the postoperative changes in the three rotational components of head orientation—yaw (transverse plane), pitch (sagittal plane), and roll (frontal plane)—each obtained from the Euler-angle decomposition of the T0–T1 postural change described above. Positive values denote, respectively, rotation about the vertical axis (yaw), upward chin rotation/head extension (pitch), and lateral tilt (roll).

### 2.7. Statistical Analysis

All analyses were performed with IBM SPSS Statistics (v29.0; IBM Corp., Armonk, NY, USA). For each rotational axis, the individual postoperative change score was calculated as Δ = T1 − T0. Distributional assumptions were assessed on the change-score distributions using the Shapiro–Wilk test. Within each skeletal class, normally distributed change scores were tested against zero using a one-sample *t*-test, which is mathematically equivalent to a paired *t*-test on T1 and T0 values; non-normally distributed changes would have been analysed with the Wilcoxon signed-rank test. Between skeletal Class II and Class III, normally distributed independent change scores were compared using Welch’s t-test because of the unequal group sizes and without assuming equal variances; non-normally distributed variables would have been compared using the Mann–Whitney U test. Results are reported as mean ± standard deviation, 95% confidence interval, *p*-value, and standardized effect size. Cohen’s dz was used for within-group change and Hedges’ g for between-group differences. A two-tailed *p* < 0.05 was considered statistically significant. Previously published NHP reproducibility ranges are discussed only as contextual information and were not used as study-specific measurement-error thresholds or as evidence of internal validation.

### 2.8. Ethical Considerations

This retrospective study was conducted in accordance with the principles of the Declaration of Helsinki. The study represents a secondary analysis of anonymized three-dimensional facial scan data collected within an approved research protocol entitled “Facial profilometric analysis: the role of artificial intelligence” (Protocol No. 189/2023; Principal Investigator: V.A.), which received a favourable opinion from the Ethics Committee Campania 3 (Comitato Etico Campania 3) on 17 April 2024 (Minutes No. 6/24; protocol registration No. 00013842 of 19 April 2024). The Ethics Committee was established by Deliberation of the Regional Council of Campania No. 224 of 27 April 2023. The approved protocol explicitly included the profilometric and three-dimensional surface analysis of the face. No additional imaging procedures or patient contact beyond the approved protocol were required.

## 3. Results

A total of 54 adult patients were included, comprising 17 patients with skeletal Class II and 37 with skeletal Class III malocclusion. The overall male-to-female ratio was 38:16 (approximately 2.4:1). All patients had paired preoperative and 6-month postoperative assessments of NHP. The unequal group sizes reflected the distribution of consecutive eligible cases. All yaw, pitch, and roll change-score distributions were approximately normal within each skeletal class (Shapiro–Wilk *p* > 0.05), supporting parametric analysis.

Within skeletal Class II (*n* = 17), the mean T0–T1 change was +2.28° ± 1.04 for yaw (95% CI 1.75 to 2.81; Cohen’s dz = 2.19; *p* < 0.001), −1.30° ± 1.34 for pitch (95% CI −1.99 to −0.61; Cohen’s dz = −0.97; *p* = 0.001), and +2.89° ± 1.21 for roll (95% CI 2.27 to 3.51; Cohen’s dz = 2.39; *p* < 0.001) (Table 1). These values describe the magnitude and direction of change within the present cohort and should not be interpreted as established thresholds of clinical importance.

Within skeletal Class III (*n* = 37), the mean T0–T1 change was +4.61° ± 1.04 for yaw (95% CI 4.26 to 4.96; Cohen’s dz = 4.43; *p* < 0.001), +1.96° ± 0.99 for pitch (95% CI 1.63 to 2.29; Cohen’s dz = 1.98; *p* < 0.001), and +4.04° ± 1.02 for roll (95% CI 3.70 to 4.38; Cohen’s dz = 3.96; *p* < 0.001) (Table 1). Pitch therefore showed an opposite mean direction of change in Class III compared with Class II.

Between-group comparisons of change scores confirmed significant differences for all three axes (Table 2). The mean Class III-minus-Class II difference was 2.33° for yaw (95% CI 1.71 to 2.95; Hedges’ g = 2.21; *p* < 0.001), 3.26° for pitch (95% CI 2.51 to 4.01; Hedges’ g = 2.90; *p* < 0.001), and 1.15° for roll (95% CI 0.46 to 1.84; Hedges’ g = 1.05; *p* = 0.002). These statistically significant differences indicate distinct class-specific patterns in the present sample; however, no clinically important angular threshold was prespecified or validated, and the measurements should not be interpreted as proof of a specific biological mechanism or clinical benefit.

## 4. Discussion

Previous literature has described associations between craniofacial morphology and head posture and has proposed that some postural patterns may be compensatory [1,7]. In the present cohort, direction-specific T0-T1 changes in NHP were observed in both skeletal Class II and Class III patients. Because this was a retrospective pre-post study without an untreated comparator, these findings should be interpreted as postoperative temporal associations rather than as evidence that surgery itself caused a specific cranio-cervical adaptation.

The opposite direction of mean pitch change between skeletal Class II and Class III was the most distinctive between-group observation. Previous literature has proposed that sagittal skeletal relationships may be accompanied by different habitual head-posture patterns [8,9,10], and such mechanisms may be compatible with the present findings. However, the current data do not establish that the observed pitch changes represent resolution of a preoperative compensation. Likewise, the observed yaw and roll differences cannot be attributed specifically to changes in occlusion, symmetry, neuromuscular activity, cervical biomechanics, airway function, splint use, or soft-tissue adaptation because these mechanisms were not directly measured. Such explanations should therefore be regarded as hypotheses for prospective investigation rather than conclusions of the present study.

Previous studies have documented postoperative head-posture changes and methodological approaches to recording or reproducing NHP using cephalometric, CBCT-based, stereophotogrammetric, and other three-dimensional techniques [8,9,10,14,15,16,17,18,19,20,21]. The incremental contribution of the present study is the simultaneous description of yaw, pitch, and roll in both skeletal Class II and Class III groups using one standardized laser-referenced acquisition and analytical workflow. The retained external Cartesian reference and the use of upper-facial landmarks facilitate objective serial comparison, but these technical features do not constitute formal validation of the complete measurement method. Published NHP reproducibility ranges obtained with other protocols may provide contextual information [3,15,16,17,21], but they cannot be treated as a study-specific measurement-error threshold or used to distinguish biological change from methodological variability in the present dataset.

From a clinical-methodological perspective, head orientation should be standardized and documented when virtual surgical plans, facial symmetry, profile projection, or airway dimensions are compared over time [4,9,22,23,24,25,26]. A change in NHP may alter the apparent roll, yaw, facial projection, and head-position-dependent upper-airway geometry without necessarily representing an equivalent skeletal or functional change. The present findings therefore support careful documentation of head orientation during image acquisition and interpretation. They do not demonstrate improved virtual-planning accuracy, airway function, facial symmetry, aesthetic outcome, or patient satisfaction, and no degree of postoperative rotation identified here can presently be recommended as a threshold for treatment modification.

Statistical significance should not be interpreted as established clinical importance. The study did not define or validate a minimal clinically important angular difference and did not include cervical-spine measures, muscle activity, respiratory or airway function, temporomandibular function, symptoms, aesthetic satisfaction, quality of life, or other patient-reported outcomes. Longitudinal studies incorporating these domains are required to determine whether the observed orientation changes persist over time and whether they are associated with clinically meaningful functional or patient-centred outcomes.

### 4.1. Limitations

Several limitations should be acknowledged. First, the single-centre retrospective pre-post design limits control over confounding and precludes causal inference. The absence of an untreated comparator means that the observed T0–T1 changes cannot be attributed exclusively to orthognathic surgery, and the absence of an independent validation cohort limits external generalizability and reproducibility across settings. Second, reliability should be considered at both acquisition and post-processing levels. Repeated complete facial acquisitions were not systematically obtained; repeated landmark-identification sessions, a second observer, and an independent reference method were not included in the retrospective protocol. Consequently, study-specific acquisition repeatability, intra- and inter-observer reliability, absolute measurement error, and criterion validity could not be directly quantified. The use of the same operator, scanner, laser reference, morning acquisition window, environmental conditions, and processing workflow represents procedural standardization but should not be interpreted as formal methodological validation.

Third, the skeletal groups were unequal in size (17 Class II and 37 Class III), reflecting the distribution of consecutive eligible cases. Although Welch’s *t*-test was used for between-group comparisons, the smaller Class II subgroup yields less precise estimates and limits subgroup generalizability; no predictive inference should be made from these data. Fourth, registration relied on manually identified soft-tissue upper-facial landmarks, which introduces a potential source of observer-related variability despite selection of regions away from the operative field. Fifth, orthodontic appliance status differed at T1 according to completion of postoperative orthodontic treatment, although the dentoalveolar region and brackets were not used as registration references or for angle calculation. Sixth, no validated clinically important threshold or functional, symptomatic, aesthetic, airway, cervical, neuromuscular, temporomandibular, or patient-reported outcome was available to establish the clinical meaning of the measured rotations. Finally, follow-up was limited to 6 months, and the long-term stability of the observed orientation changes remains unknown.

### 4.2. Future Directions

Prospective validation studies should assess the complete measurement chain using systematically repeated NHP acquisitions, repeated landmark identification, multiple observers, absolute-error and agreement analyses, and comparison with an established reference method. Larger and more balanced cohorts with independent validation samples and longer follow-up are required to assess external reproducibility. Future work should also investigate associations between three-dimensional head orientation and cervical-spine biomechanics, neuromuscular function, airway and respiratory measures, temporomandibular function, facial symmetry, aesthetic outcomes, and patient-reported measures. Automated posture analysis and geometric deep-learning methods may ultimately reduce observer dependence, but their performance should likewise be evaluated against appropriate reference standards [20,27,28].

## 5. Conclusions

At six months after bimaxillary orthognathic surgery, this retrospective cohort demonstrated direction-specific T0–T1 changes in three-dimensional head orientation in both skeletal Class II and Class III patients. The principal between-group finding was the opposite direction of mean pitch change, together with larger mean yaw and roll changes in the Class III group. Because the study did not include repeated complete acquisitions, study-specific observer-reliability and measurement-error analyses, an untreated comparator, an independent validation cohort, or functional and patient-reported outcomes, the findings should be interpreted as exploratory measurements rather than as evidence of physiological normalization, neuromuscular adaptation, or clinical benefit. The laser-referenced three-dimensional workflow provides a standardized means of expressing serial head orientation relative to an external Cartesian reference; however, prospective validation with repeated acquisitions, multiple observers, absolute-error assessment, and comparison with an established reference method is required before the approach can support predictive or patient management recommendations.

## Figures and Tables

**Table 1:** Within-class postoperative rotational change (Δ = T1 − T0).

Parameter	Class	N	Mean ± SD (°)	95% CI (°)	Cohen’s dz	*p*-Value
Yaw	II	17	2.28 ± 1.04	1.75 to 2.81	2.19	<0.001
Yaw	III	37	4.61 ± 1.04	4.26 to 4.96	4.43	<0.001
Pitch	II	17	−1.30 ± 1.34	−1.99 to −0.61	−0.97	0.001
Pitch	III	37	1.96 ± 0.99	1.63 to 2.29	1.98	<0.001
Roll	II	17	2.89 ± 1.21	2.27 to 3.51	2.39	<0.001
Roll	III	37	4.04 ± 1.02	3.70 to 4.38	3.96	<0.001

Δ, individual T1 − T0 change score; CI, confidence interval; SD, standard deviation; Cohen’s dz, standardized within-group effect size.

**Table 2:** Between-class comparison of postoperative rotational change.

Parameter	Class II Mean ± SD (°)	Class III Mean ± SD (°)	Difference III–II (°)	95% CI of Difference (°)	Hedges’ g	*p*-Value
Yaw	2.28 ± 1.04	4.61 ± 1.04	2.33	1.71 to 2.95	2.21	<0.001
Pitch	−1.30 ± 1.34	1.96 ± 0.99	3.26	2.51 to 4.01	2.90	<0.001
Roll	2.89 ± 1.21	4.04 ± 1.02	1.15	0.46 to 1.84	1.05	0.002

Between-class *p*-values correspond to Welch’s *t*-test. CI, confidence interval; SD, standard deviation; Hedges’ g, bias-corrected standardized between-group effect size. Positive differences denote greater mean change in Class III.

## Data Availability

The data presented in this study are available on reasonable request from the corresponding author. The data are not publicly available owing to privacy and ethical restrictions.

## References

[1] Moorrees C.F.A., Kean M.R. (1958). Natural head position, a basic consideration in the interpretation of cephalometric radiographs. Am. J. Phys. Anthropol..

[2] Lundström F., Lundström A. (1992). Natural head position as a basis for cephalometric analysis. Am. J. Orthod. Dentofac. Orthop..

[3] Madsen D.P., Sampson W.J., Townsend G.C. (2008). Craniofacial reference plane variation and natural head position. Eur. J. Orthod..

[4] Bansal N., Singla J., Gera G., Gupta M., Kaur G. (2012). Reliability of natural head position in orthodontic diagnosis: A cephalometric study. Contemp. Clin. Dent..

[5] Meiyappan N., Tamizharasi S., Senthilkumar K.P., Janardhanan K. (2015). Natural head position: An overview. J. Pharm. Bioallied Sci..

[6] Kadhom Z.M., Jumaa N. (2020). Natural head position: A review. J. Baghdad Coll. Dent..

[7] Solow B., Tallgren A. (1976). Head posture and craniofacial morphology. Am. J. Phys. Anthropol..

[8] Kim D.S., Yang H.J., Huh K.H., Lee S.S., Heo M.S., Choi S.C., Hwang S.J., Yi W.J. (2014). Three-dimensional natural head position reproduction using a single facial photograph based on the POSIT method. J. Craniomaxillofac. Surg..

[9] Hsung T.C., Lo J., Li T.S., Cheung L.K. (2014). Recording of natural head position using stereophotogrammetry: A new technique and reliability study. J. Oral Maxillofac. Surg..

[10] Cho D., Choi D.S., Jang I., Cha B.K. (2015). Changes in natural head position after orthognathic surgery in skeletal Class III patients. Am. J. Orthod. Dentofac. Orthop..

[11] Liu X.J., Li Q.Q., Pang Y.J., Tian K.Y., Xie Z., Li Z.L. (2015). Modified method of recording and reproducing natural head position with a multicamera system and a laser level. Am. J. Orthod. Dentofac. Orthop..

[12] Lin X. (2024). Orthognathic surgery improves compromised natural head position and pharyngeal airway in patients with skeletal Class II or III malocclusion. J. Oral Rehabil..

[13] Zhu S., Keeling A., Hsung T.C., Yang Y., Khambay B. (2018). The difference between registered natural head position and estimated natural head position in three dimensions. Int. J. Oral Maxillofac. Surg..

[14] Yang H.J., Han J.J., Hwang S.J. (2018). Accuracy of 3D reproduction of natural head position using three different manual reorientation methods compared to 3D software. J. Craniomaxillofac. Surg..

[15] Demétrio M.S., Marlière D.A.A., Barbosa S.M., Pereira R.A., da Silveira H.M. (2021). Different modalities to record and transfer natural head position to virtual planning in orthognathic surgery: Case reports of asymmetric patients. J. Maxillofac. Oral Surg..

[16] Lee S.J., Yoo J.Y., Woo S.Y., Yang H.J., Kim J.E., Huh K.H., Lee S.S., Heo M.S., Hwang S.J., Yi W.J. (2021). A complete digital workflow for planning, simulation, and evaluation in orthognathic surgery. J. Clin. Med..

[17] Al-Yassary M., Billiaert K., Antonarakis G.S., Kiliaridis S. (2022). Evaluation of natural head position over five minutes: A comparison between an instantaneous and a five-minute analysis with an inertial measurement unit. J. Oral Rehabil..

[18] Beek D.M., Baan F., Liebregts J., Bergé S., Maal T., Xi T. (2022). Reproducibility of manual transfer of the clinical natural head position: Influence on the soft tissue and hard tissue position of three-dimensional virtual surgical planning. J. Oral Maxillofac. Surg..

[19] Sánchez C.A.F., Laverde C.O.D., Rodríguez S.E.N., Gallego G.A.C., Aristizábal J.F., Salazar O.I.C. (2024). Methodology for the correction of a CBCT volume from the skull to the natural head position. MethodsX.

[20] Yoo J.Y., Yang S., Lim S.H., Han J.Y., Kim J.M., Kim J.E., Huh K.H., Lee S.S., Heo M.S., Yang H.J. (2025). Automatic reproduction of natural head position in orthognathic surgery using a geometric deep learning network. Diagnostics.

[21] Cassi D., De Biase C., Tonni I., Gandolfini M., Di Blasio A., Piancino M.G. (2016). Natural position of the head: Review of two-dimensional and three-dimensional methods of recording. Br. J. Oral Maxillofac. Surg..

[22] Alkaabi S., Maningky M., Helder M.N., Alsabri G. (2022). Virtual and traditional surgical planning in orthognathic surgery: Systematic review and meta-analysis. Br. J. Oral Maxillofac. Surg..

[23] Strujak G., Marlière D.A.A., Medeiros Y.L., Guariza Filho O., Carlini J.L., Westphalen V.P.D. (2024). Virtual versus conventional planning in orthognathic surgery: A systematic review and meta-analysis. J. Maxillofac. Oral Surg..

[24] Hernández-Alfaro F., Giralt-Hernando M., Brabyn P.J., Haas O.L., Valls-Ontañón A. (2021). Variation between natural head orientation and Frankfort horizontal planes in orthognathic surgery patients: 187 consecutive cases. Int. J. Oral Maxillofac. Surg..

[25] Akhlaghi F., Torabi Z.S., Tabrizi R. (2025). Changes in lateral standing posture following orthognathic surgery: A cohort study. Int. J. Oral Maxillofac. Surg..

[26] Swennen G.R.J., Mollemans W., De Clercq C., Abeloos J., Lamoral P., Lippens F., Neyt N., Casselman J., Schutyser F. (2009). A cone-beam computed tomography triple scan procedure to obtain a three-dimensional augmented virtual skull model appropriate for orthognathic surgery planning. J. Craniofac. Surg..

[27] Alexa V.T., Fratila A.D., Szuhanek C., Jumanca D., Lalescu D., Galuscan A. (2022). Cephalometric assessment regarding craniocervical posture in orthodontic patients. Sci. Rep..

[28] Millesi G.A., De Fazio G.R., Zimmermann M., Eltz M. (2025). Improved stability of open bite deformities: Taking control of the transverse width using digital technology and robotic archwire bending. Int. J. Oral Maxillofac. Surg..

